# Exploring the Anticancer Potential of Schiff Base Complexes: A Mini‐Review

**DOI:** 10.1002/cmdc.70484

**Published:** 2026-09-09

**Authors:** Atif Ahmad, Anam, Marcos V. Palmeira‐Mello, Sabir Khan, José Clayston Melo Pereira

**Affiliations:** ^1^ Departamento de Química Analítica, Físico‐Química e Inorgânica Instituto de Química, UNESP – Universidade Estadual Paulista Araraquara Brazil; ^2^ Departamento de Química Universidade Federal de São Carlos (UFSCar) São Carlos Brazil; ^3^ Universidade Federal de Pelotas (UFPel) Pelotas Brazil

**Keywords:** cytotoxicity on tumor cell lines, DNA binding modes, inducing cell death mechanisms, Schiff base metal complexes

## Abstract

Schiff bases are well known for their ability to form stable coordination compounds with several transition metals, making them valuable for medicinal chemistry purposes. These metal‐based compounds have attracted significant attention due to their enhanced biological activities, particularly their ability to act toward anticancer chemotherapy. This mini‐review provides a comprehensive overview of Schiff base complexes reported over the last 5 years, highlighting the recent advances related to their biological investigations. Cytotoxicity and interactions with biological targets have been detailed. The mechanisms underlying the capacity of these complexes to induce cell death in cancer cells are also described.

## Graphical abstract

Schiff base metal complexes as promising anticancer agents: highlighting DNA interactions, cytotoxicity, molecular targets, and mechanisms of cancer cell death.

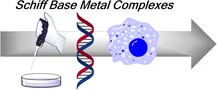

**Abbreviations**
5‐FU: 5‐fluorouracil
A549: human lung tumor cells
AGS: human stomach tumor cells
ALL: acute lymphoblastic leukemia
AML: acute myeloid leukemia
ATase: amidophosphoribosyltransferase
CML: chronic myeloid leukemia
CT‐26: mouse colon cancer cells
DHFR: dihydrofolate reductase
DLD‐1: human colorectal cancer cell
DNA: deoxyribonucleic acid
DPPH 2: 2,2‐diphenyl‐1‐picrylhydrazyl
Eca‐109: human esophageal squamous cancer cells
FDA: Food and Drug Administration
GARFT: glycinamide ribonucleotide formyltransferase
HepG2: human liver tumor cells
HCT‐116: human colon tumor cells
HT‐29: human colon tumor cells
HeLa: human cervical tumor cells
HEK293: human normal cells of embryonic kidney
IC_50_
: half maximal inhibitory concentration
MCF‐7: human breast tumor cells
MDA‐MB‐231: human triple‐negative breast tumor cells
MTT 3‐(4,5‐dimethylthiazol‐2‐yl)‐2: 3‐(4,5‐dimethylthiazol‐2‐yl)‐2,5‐diphenyltetrazolium bromide
NIH/3T3 cells: normal cell lines derived from mouse fibroblast
PC3: human prostate tumor cells
ROS: reactive oxygen species
TS: thymidylate synthase
WI38: human normal cells of lung fibroblast
xCT: cystine/glutamate transporter
XRD: X‐ray diffraction

## 1 Introduction

Cancer, a term encompassing over 100 diseases, is characterized by the growth of irreversibly mutated cells that multiply beyond their usual boundaries in the body, forming a tissue mass known as a tumor [1, 2, 3, 4]. Tumor cells have distinct morphologies in comparison to healthy cells [5]. Furthermore, they lack the process of differentiation, which leads to more genetic mutations and rapid proliferation [5]. These cells can spread to other parts of the body, in a process called metastasis [2, 3]. Carcinogenesis, e.g., the tumor formation process, depletes the body's resources of oxygen and nutrients [6], weakening the immune system [7] and causing other disruptions that hinder normal bodily functions. The most prevalent types include breast, lung, colorectal, and prostate cancers [3, 8]. Behavioral and dietary factors, physical inactivity, tobacco, and alcohol use contribute significantly to cancer mortality, as well as environmental exposures to carcinogens such as UV radiation, asbestos, and contaminated water [2, 9, 10].

As a public health challenge, cancer accounted for nearly 19.3 million new cancer cases and 10 million deaths worldwide in 2020 [3, 11, 12]. In 2023, approximately 1.96 million new cases were reported only in the United States of America (USA), which represents around 5,370 cases daily [8]. Likewise, an estimation of 1,670 daily fatalities were reported in the same period [8], whereas Han et al. [13] estimated 2,574,200 deaths in 2022 in China. Cancer mortality is projected to rise to 2.1 million by 2030 in the Americas [2]. It is noteworthy that low‐ and middle‐income countries face higher incidence and mortality of cancer, owing to approximately 70% of deaths occur in these regions [2]. Limited access to diagnostic and treatment resources exacerbates these disparities.

Cancer is not only a significant public health issue but also a major economic burden globally. The economic implications extend beyond healthcare costs to include lost labor productivity and depleted savings. Estimates project that without increased investment in research and prevention, cancer will cost the global economy an accumulated Int$25.2 trillion over the next 30 years [14].

Recently, cancer diagnosis and treatment have been delayed due to the COVID‐19 pandemic overwhelming the health system [8]. This has likely led to a backlog of undiagnosed cases and could result in patients presenting with more advanced stages of disease [15].

Concern about this disease and the damage it inflicts on society has led researchers to develop various treatments, including immunotherapy, photodynamic therapy, surgery, and chemotherapy [4, 12, 16]. In the context of chemotherapy, platinum(II) complexes, such as cisplatin, carboplatin, and oxaliplatin, have been used as metallodrugs in 50% of the cases [4, 17, 18].

### 1.1 List of Non‐Platinum Approved Drugs

**FIGURE 1:**
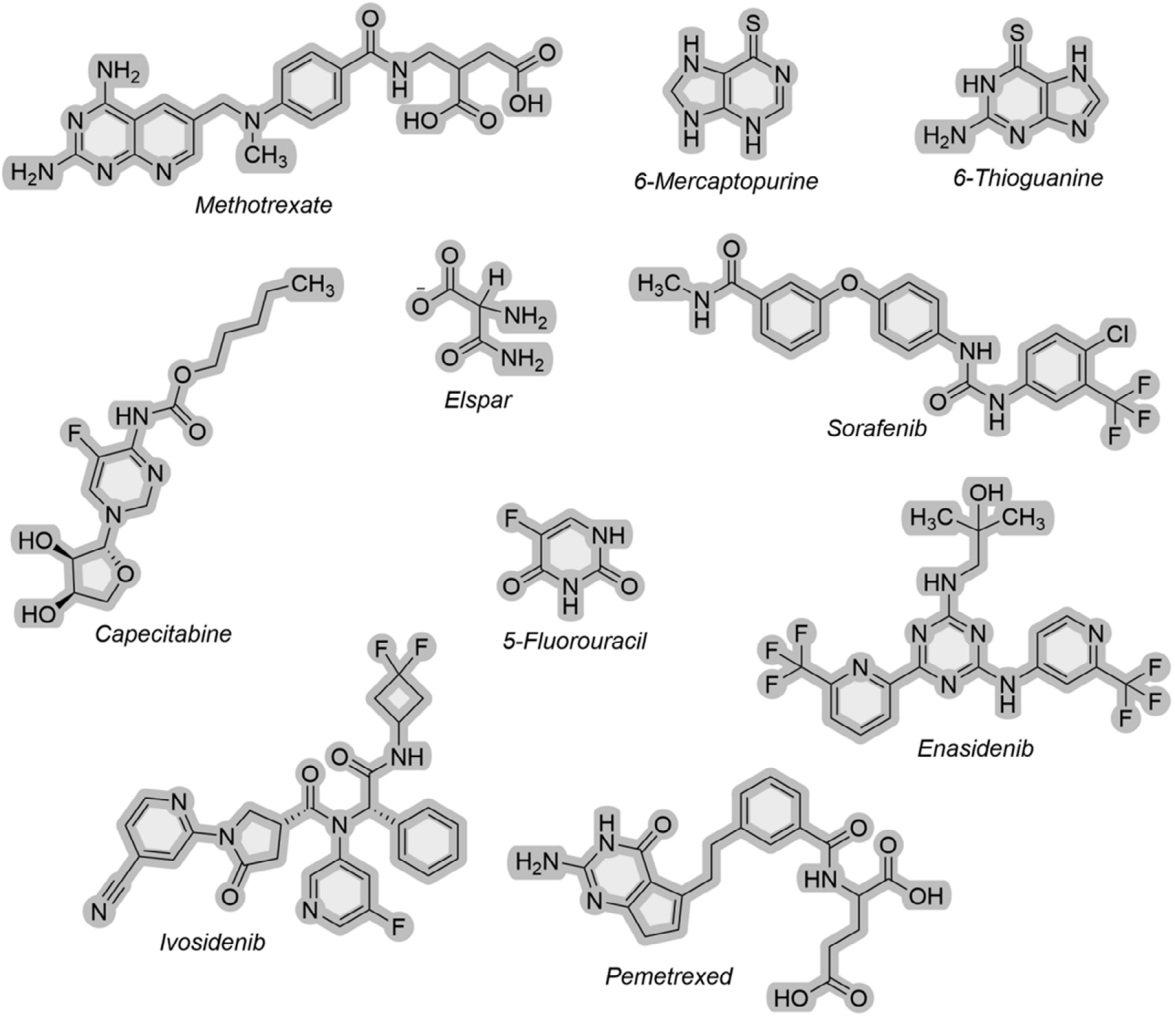
Chemical structures of non‐platinum FDA‐approved anticancer drugs.

The non‐platinum drugs approved by the Food and Drug Administration (FDA) are shown in Table 1, and their structures are shown in Figure 1.

**TABLE 1:** List of non‐platinum FDA‐approved drugs.

Drugs names	Year of approval	Action	Tumor cells
Methotrexate	1953	DHFR	Breast cancer, epidermoid cancers of the head and neck, cutaneous T cell lymphoma, lung cancer, etc.
6‐Mercaptopurine	1953	ATase	ALL
6‐Thioguanine	1966	ATase	AML, ALL, and CML
Elspar	1978	Asparagine	ALL
Capecitabine	1998	TS	Colorectal cancer and breast cancer
5‐Fluorouracil	2000	TS	Colorectal cancer and other gastrointestinal cancers, breast cancer, neuroendocrine tumors, thymic cancer, cervical cancer, bladder cancer, hepatobiliary cancer
Pemetrexed	2004	DHFR, TS, GARFT	Malignant pleural mesothelioma, lung cancer
Sorafenib	2005	Many protein kinases, xCT	Renal cell carcinoma, hepatocellular carcinoma, and thyroid cancer
Enasidenib	2017	IDH2 mutated	AML
Calaspargase pegol‐mknl	2018	Asparagine	ALL
Ivosidenib	2019	IDH1 mutated	AML

### 1.2 Platinum‐Based Compounds as Anticancer Agents

**FIGURE 2:**
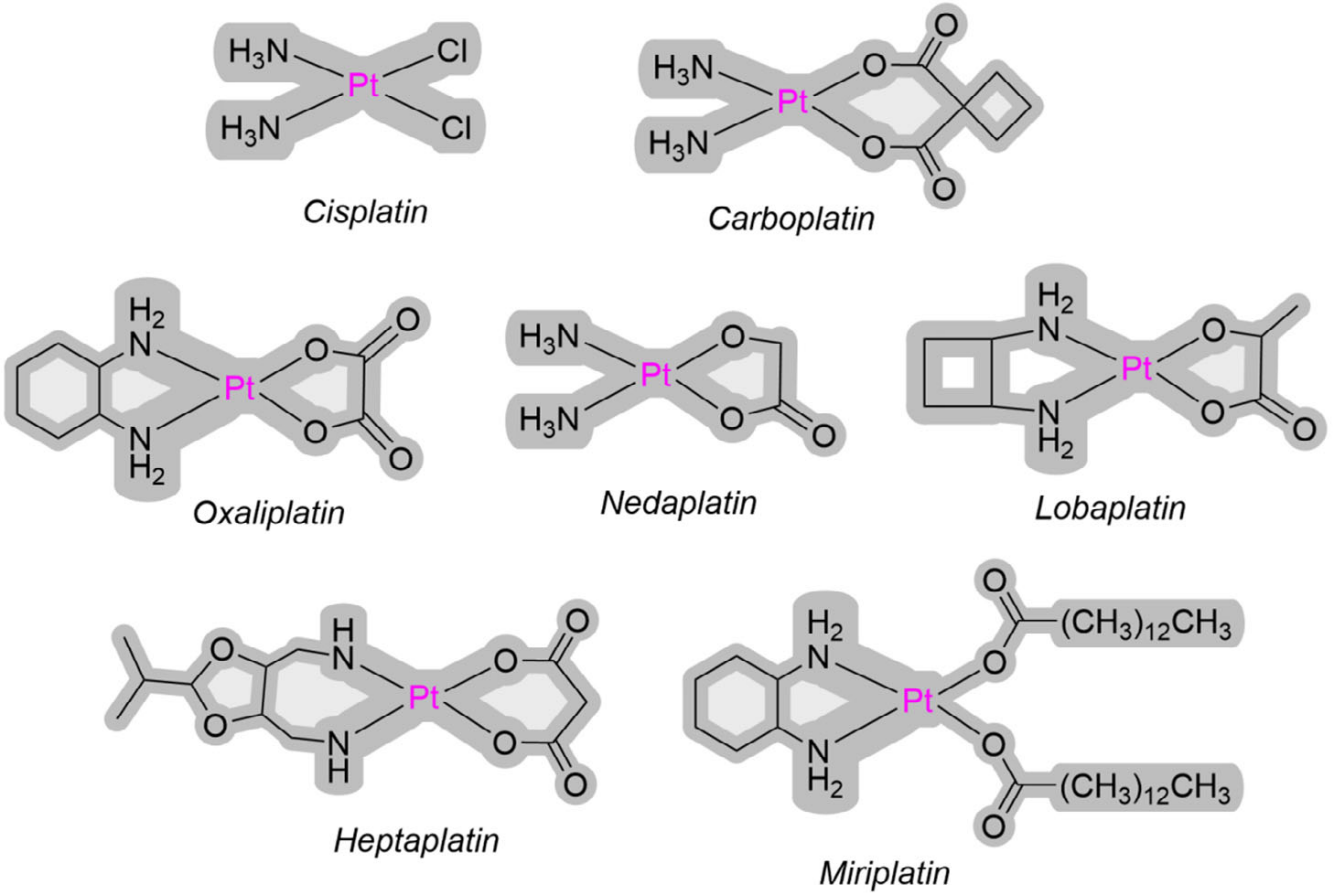
Platinum (II)‐based FDA‐approved chemotherapeutic drugs.

Chemotherapy is a primary approach used for cancer treatment when it is diagnosed at an early stage [19]. In 1965, cisplatin (*cis*‐[PtCl_2_(NH_3_)_2_]) was reported to have antitumor properties by Rosenberg [17]. It effectively inhibited the cell division of *Escherichia coli*, as well as the cell lines Sarcoma‐180 and L1210 (leukemia), suppressing tumors in vivo [20]. It was subsequently approved by the FDA in 1978 [20]. This highlights the important role of inorganic substances in cancer treatment. Platinum(II)‐based anticancer drugs are widely used worldwide to treat various types of cancer, including oxaliplatin, carboplatin, lobaplatin (China), heptaplatin (Korea), nedaplatin, and miriplatin (Japan) (Figure 2) [21, 22, 23, 24].

Cisplatin exerts its anticancer effects primarily through either genomic DNA (gDNA) or mitochondrial DNA (mDNA) damage mechanisms [4]. Once inside the cell, cisplatin undergoes activation by aquation, where one of its chloride ligands is replaced by a water molecule due to the lower chloride concentration in the cytoplasm [25, 26]. This activated form of cisplatin binds covalently to the N7 position of purine bases in DNA, especially via guanine, forming inter and intra cross‐links [25, 27, 28]. These lesions disrupt DNA replication and transcription, triggering apoptosis [25, 26, 27, 29, 30]. However, its non‐specificity toward cancerous versus normal cells contributes to significant side effects such as nausea, nephrotoxicity, neurotoxicity, and ototoxicity [29, 30, 31, 32].

One of the challenges in the clinical application of cisplatin is the development of drug resistance, which can occur via mechanisms such as reduced drug uptake, enhanced DNA repair, and drug inactivation by cellular proteins [4, 17, 28, 30]. Additionally, the high systemic toxicity and side effects have driven efforts to design and synthesize analogues with improved therapeutic indexes. Carboplatin, for instance, features a bis‐carboxylate ligand that slows its aquation rate, reducing toxicity while maintaining efficacy [29, 30]. However, cell resistance is a massive problem for carboplatin, especially in ovarian cancer cases [33].

Despite its limitations, cisplatin remains a cornerstone of chemotherapy. Research continues to explore novel platinum‐based compounds, focusing on overcoming resistance and minimizing toxicity by leveraging structure–activity relationship studies [34]. For example, strategies include modifying ligands to alter reactivity and designing prodrugs to enhance tumor selectivity. These advancements underscore the enduring importance of cisplatin in cancer treatment and the ongoing innovation in platinum‐based chemotherapy [34].

Such findings have opened new opportunities for research in the development of metal‐based therapeutic drugs, which exhibit different kinetics and mechanisms of action compared to conventional organic agents [23, 35].

### 1.3 General Structure and Medicinal Importance of Schiff Bases

**SCHEME 1:**
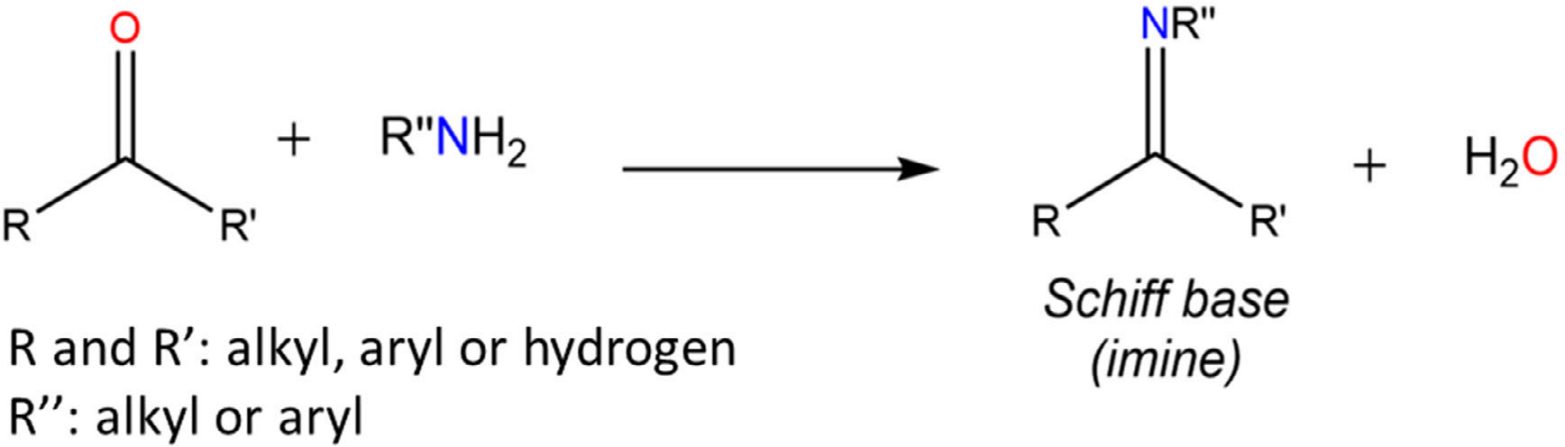
General reaction of Schiff base formation.

The concept of Schiff bases was discovered in 1864 by Hugo Schiff, whose was later awarded the Nobel Prize for his work [21]. Schiff bases are organic compounds that typically contain an azomethine or imine group [36, 37, 38]. The general chemical structure of a Schiff base is shown in Scheme 1. The sp^2^‐hybridized nitrogen atom in the azomethine linkage contains a lone pair of electrons, which plays a crucial role in exhibiting biological activity [39] (Scheme 1), which confers the C=N moiety as the primarily responsible for their diverse biological activities [39, 40]. The Schiff condensation involves the reaction of a carbonyl compound with an amine, yielding Schiff bases with high yields [41]. The mechanism for this process is typically synthesized through an acid‐catalyzed reaction [42]. Schiff bases and their metal complexes have been explored due to their several properties such as antimicrobial, antileishmanial, anti‐inflammatory, anti‐*Mycobacterium*, antioxidant, and anticancer [43].

### 1.4 Applications of Schiff Base Complexes

Schiff bases play a crucial role in coordination chemistry due to their ability to form stable metal complexes [18, 44, 45, 46]. These ligands coordinate metal ions via nitrogen and additional donor atoms such as oxygen, sulfur, or phosphorus, leading to diverse geometries like octahedral, square planar, and tetrahedral [18, 38, 47]. The versatility of Schiff base metal complexes has led to their extensive study in catalysis [36], bioinorganic chemistry [48], and materials science [49], in which they serve as efficient catalysts in oxidation and polymerization reactions [50]. Their biological properties [51, 52, 53, 54, 55] include antibacterial [52, 56, 57], antifungal [52, 57, 58, 59], anti‐Alzheimer's [60], antituberculosis [61], antioxidant [62], antileishmanial [43], antimicrobial [63], anti‐inflammatory [64], anti‐glycation [43], urease inhibitors [43], and anticancer [65, 66, 67]. This effect may be due to their planar structure that allows strong interactions with biomolecules like DNA and proteins [55, 68]. The presence of the azomethine group enhances binding affinity, making these complexes promising candidates for medicinal applications [69]. Moreover, their remarkable selectivity, sensitivity, and stability toward metal ions make them highly effective in metal chelation and complex stabilization [70]. Ongoing research focuses on optimizing Schiff base ligand structures and exploring new metal combinations to enhance stability [70], selectivity [71], and functional properties, further expanding their potential in drug development [72].

### 1.5 DNA‐Interacting Properties and Mechanism of Action

DNA remains as the main biological target for different bioactive molecules [73] Schiff bases and their metal complexes interact with DNA [18, 46, 74] through various mechanisms, including intercalation [75], groove binding [76], and electrostatic interactions [77]. The interactions between metal complexes bearing Schiff bases and DNA can lead to structural distortions in the biomolecule, affecting its structure, potentially inhibiting replication and transcription processes, leading to cell death [78].

The structure of Schiff bases enables intercalation [79], in which the ligand inserts itself between the stacked base pairs of DNA [79], stabilizing the complex through π–π stacking interactions and disrupting the DNA helix [80]. Additionally, these species can bind within the minor or major grooves of DNA through hydrogen bonding, van der Waals forces, and hydrophobic interactions [81]. The azomethine nitrogen (—C=N—) and other donor atoms in the Schiff base ligand play a key role in forming hydrogen bonds with the DNA bases or the sugar–phosphate backbone, enhancing binding specificity and affinity [74]. As phosphates are negatively charged groups, the positive charge of the metal center in these complexes may facilitate the interaction via electrostatic forces, further stabilizing the DNA–complex interaction [71]. These characteristics make the Schiff bases and their complexes promising candidates for applications for DNA‐targeted therapies in anticancer research [82, 82]. In general, the specific mechanism of interaction depends on the structural characteristics of the Schiff base ligand, the nature of the metal ion, and the overall geometry of the complex [83].

These complexes mainly induce apoptosis via both intrinsic and extrinsic pathways [46, 84]: The intrinsic pathway is triggered when metal complexes generate oxidative stress, causing mitochondrial membrane depolarization, cytochrome c release, and sequential activation of caspase‐9 and caspase‐3 [85], while the extrinsic pathway is initiated through death receptor activation and caspase‐8 mediation [86]. Additionally, certain Schiff base complexes can induce necrosis by producing excessive reactive oxygen species (ROS), leading to rapid plasma membrane rupture, cellular swelling, and inflammatory responses [87]. The lipophilicity of these complexes further enhances their membrane permeability, allowing direct organelle targeting and DNA damage. Notably, some Schiff base complexes exhibit dual functionality, which makes them effective against resistant cancer cells, specifically by activating apoptotic pathways and inducing necrosis‐like membrane rupture. This multimodal mechanism explains the observed efficiency of Schiff base metal complexes in overcoming conventional chemotherapy limitations.

## 2 Schiff Bases Complexes as Anticancer Agents

**FIGURE 3:**
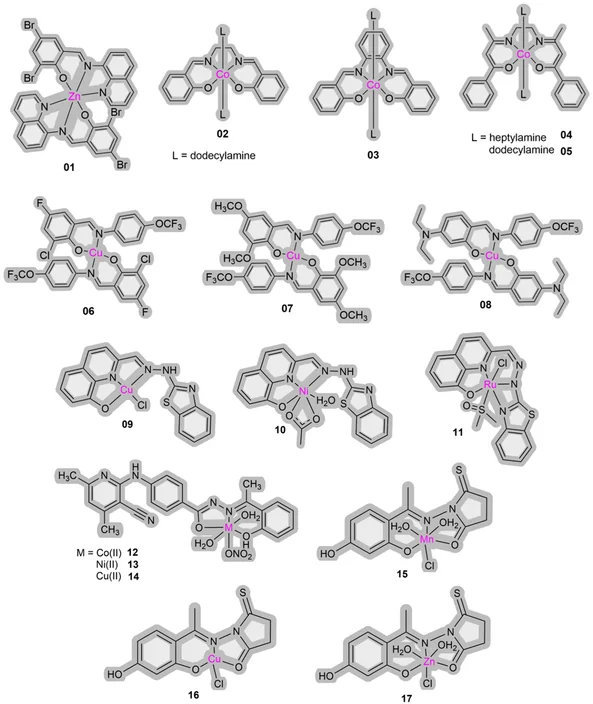
Schiff base complexes investigated as anticancer agents.

Several researchers have made an effort to obtain new Schiff base metal complexes as anticancer agents. Pinchaipat et al. [88] reported the synthesis of a new Schiff base Zn(II) complex (**01**) (Figure 3). This compound was characterized by ultraviolet–visible (UV–vis) and Fourier transform infrared (FT‐IR) spectroscopies, and ^1^H‐NMR (nuclear magnetic resonance) and mass spectrometry. In addition, its solid structure was studied by X‐ray diffraction (XRD). The anticancer activity of **01** was evaluated against A549 cancer cells via MTT assay. The zinc(II) complex exhibited moderate activity, with an IC_50_ value of 122.83 ± 4.50 µM, compared to the drug cisplatin (IC_50_ = 71.02 µM). The authors performed a DNA binding study of this complex using spectrophotometric titration, and fluorescence competitive assay using ethidium bromide (EB). The results revealed a binding constant (*K*_b_) of 2.00 × 10^6^ M^–1^, and a *K*_q_ equal to 7.91 × 10^11^ M^–1 ^s^–1^, suggesting that the planar structure of the Zn(II) complex allows its intercalation between two adjacent DNA base pairs.

Gowdhami et al. [32] synthesized two different Co(III) complexes **02** and **03** containing salen ligands (Figure 3) and evaluated their anticancer properties toward A549, MCF‐7, and 3T3 tumor cells at different times of incubation. These complexes exhibited increased cytotoxicity in a time‐dependent manner, with best performance against breast cancer cells. The IC_50_ values obtained for these compounds after 72 h of treatment were 8.56 ± 0.07 and 3.55 ± 0.08 µM, respectively, in MCF7 cells, compared to 19.53 ± 0.06 and 10.78 ± 0.18 µM in A549 cells. In contrast, cisplatin, a drug reference, exhibited higher IC_50_ values of 12.47 ± 0.04 and 22.38 ± 0.21 µM, for these same cells. The mechanistic studies indicated that both complexes induced apoptosis in MCF‐7 and A549 cells upon disruption of mitochondrial membrane potential (MMP) and ROS generation. The authors revealed that both complexes caused DNA damage as evidenced by comet assay. Additionally, immunofluorescence data have shown that BCL‐2 protein expression decreased in these cells, whereas BAX protein expression increased, which is in agreement with the apoptotic pathway described. In this scenario, cytochrome c could be released from mitochondria as a consequence of BAX upregulation, which triggers caspases cascade.

Gowdhami et al. [89] continued their studies involving the salen scaffold. In the same direction, the authors investigated two Co(III) complexes **04** and **05** (Figure 3) and investigated their cytotoxic potential in the same cells. Their findings revealed a similar pattern compared to the previous cobalt analogous compounds. The best performance has been obtained for MCF‐7 and comparison to A549 cancer cells. Also, these compounds act in a time‐dependent manner, presenting higher selectivity indexes. Considering MCF‐7 cancer cells, the authors obtained IC_50_ values in a range from 9.6 to 2.9 μM (complex **04**) and from 5.4 to 1.6 μM (complex **05**). Both complexes showed significantly greater potency than cisplatin (IC_50 _= 36.8 to 11.6 μM). DNA binding studies revealed binding constants (*K*_b_) around 10^3^ M^−1^, probably driven by hydrophobic interactions. Complexes **04** and **05** induced apoptosis in both cell lines through multiple targets, including DNA damage, disruption of MMP, and ROS generation, leading to modulation of apoptotic gene expression.

Schiff base copper complexes have also their behavior investigated toward cancer cells. Daravath et al. [90] studied three Cu(II) complexes (**06**, **07**, and **08**) (Figure 3) and evaluated their cytotoxicity against A549 and MCF‐7 cancer cell lines. The results revealed a slightly potent activity against MCF‐7 compared to A549 cells, with IC_50_ values in a range between 34–45  and 38–48 μM for these complexes, respectively. DNA‐interacting studies were first performed via spectrophotometric UV–vis titration and competitive fluorescence assays. UV–vis experiments revealed a high hypochromism effect (55%–70%), indicating the strong interaction between these species and the CT‐DNA. The presence of these compounds caused a remarkable fluorescence quenching of EB–DNA, indicating that these complexes can replace EB (ethidium bromide) from the biomolecule, thus revealing their intercalative binding mode. In a second part of the study, the hydrolytic cleavage potential of these compounds was confirmed by agarose gel electrophoresis using a pBR322 plasmid DNA. The authors suggest that Cu^2+^ acts as a Lewis acid promoving the DNA cleavage via nucleophilic reaction with the phosphodiester groups. Furthermore, the antioxidant potential of the complexes was confirmed by 2,2‐diphenyl‐1‐picrylhydrazyl (DPPH) assay. The scavening activity of these compounds increased in a concentration‐dependent manner, revealing 06 as a better antioxidant than **07** and **08**.

Cu(II), Ni(II), and Ru(II) complexes (**09–11**) (Figure 3) were investigated by Ribeiro et al. [91]. The in vitro anticancer activity of these Schiff base complexes was evaluated against HCT‐116 and CT‐26 cell lines, with 5‐fluorouracil (5‐FU) as a standard reference drug. The Ni(II) complex **10** exhibited IC_50_ values of 11.0 ± 2.3 μM (CT‐26) and 8.6 ± 2.0 μM (HCT‐116), while the Ru(II) complex **11** showed IC_50_ values of 21.0 ± 4.2 μM (CT‐26) and 16.6 ± 3.9 μM (HCT‐116), demonstrating significant activity against both cell lines. In contrast, the Cu(II) complex **09** was inactive compared to 5‐FU. The authors also investigated the antimigratory potential of these species via wound‐healing assay in both CT‐26 and HCT‐116 cells. Although no antimigration effect was observed by these complexes, **10** and **11** were able to inhibit the wound closure up to 22 h period. In order to obtain fresh insight regarding the mechanism of these complexes, the authors performed a cell cycle analysis. The induction of cell cycle arrest is an important hallmark used to understand the mechanism of action for several anticancer metal‐based compounds. Whereas **10** affect the cell cycle of CT‐26 cells in a concentration‐dependent manner, **11** also arrested the cycle progression in HCT‐116 cells. Overall, the authors suggest that both complexes could be further studied as an alternative to colorectal cancer treatment.

In the same direction, El‐Gammal et al. [92] reported the synthesis and characterization of Co(II), Ni(II), and Cu(II) complexes (**12**–**14**) (Figure 3)**.** The anticancer activity of these complexes was evaluated against MCF‐7 and HepG‐2 cell lines. All three complexes exhibited notable cytotoxic effects, with the Cu(II) complex **14** demonstrating superior activity, with IC_50_ values of 4.36 ± 0.6 μM (MCF‐7) and 3.18 ± 0.8 μM (HepG2) compared to colchicine control (IC_50_ = 42.06 ± 0.05 and 25 ± 0.05 μM, respectively). These results suggest that **14** exhibits superior anticancer potential toward both MCF‐7 and HepG‐2 cancer cell lines.

Mahdy et al. [93] also evaluated the cytotoxic potential of Mn(II), Cu(II), and Zn(II) complexes **15**–**17** (Figure 3) against cancer cells. In vitro experiments were performed in HepG2 and HCT‐116 cell lines, using vinblastine (IC_50_ = 8.42 ± 0.93 μM) as the reference drug. Against HepG2, the complexes exhibited cytotoxic activity in the order of increasing potency: Mn(II) (**15**) < vinblastine < Cu(II) (**16**) < Zn(II) (**17**). Conversely, against HCT‐116, the trend was Mn(II) (**15**) < vinblastine < Zn(II) (**17**) < Cu(II) (**16**), indicating that the Cu(II) complex was the most effective against HCT‐116 cell lines. In contrast, the Zn(II) complex **17** showed the highest activity against HepG‐2 tumor cells.

**FIGURE 4:**
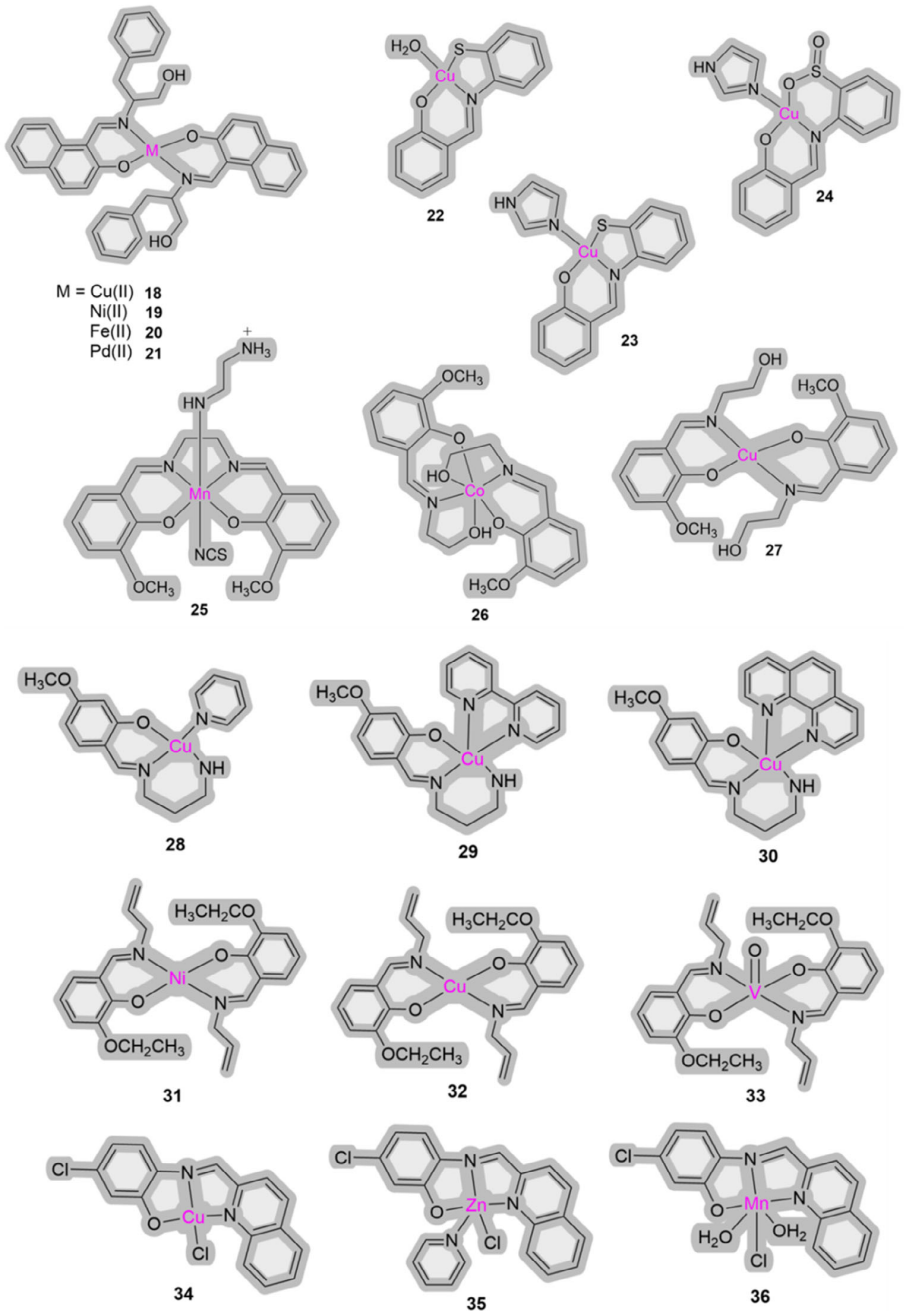
Schiff base complexes investigated as anticancer agents.

The effect of different metal ions over the coordination to a Schiff base was explored by Basaran et al. [94]. The authors synthesized and characterized four different complexes: Pd(II) (**21**), Fe(II) (**20**), Ni(II) (**19**), and Cu(II) (**18**) (Figure 4). The anticancer activity of these compounds was evaluated in vitro against DLD‐1 and MDA‐MB‐231 cell lines. Among the tested complexes, the Pd(II) complex **21** demonstrated the highest cytotoxicity, exhibiting IC_50_ values of 9.97 µM (DLD‐1) and 4.07 µM (MDA‐MB‐231), superior potency compared to cisplatin (IC_50_ = 73.2 µM). These findings suggest that the Pd(II) **21** Schiff base complex could be a promising candidate for further anticancer drug development.

Three Cu(II) complexes **22**, **23**, and **24** (Figure 4) were characterized and studied by Paliwal et al. [95]. Preliminary studies by UV–vis titration revealed that **23** and **24** bind to DNA with moderate binding constants (*K*_b_), 3.03 × 10^4^ and 3.78 × 10^4^ M^−1^, respectively. To further evaluate the main binding modes between these species, additional techniques were performed. The effect of both complexes was assessed via the relative specific viscosity (*η*/*η*_0_)^1/3^ of the DNA solution in the presence of the metal complexes. An increased viscosity profile was observed upon increasing the concentration of both compounds, indicating that **23** and **24** may intercalate between the DNA base pairs. Additionally, electrophoresis assays revealed both complexes as efficiently DNA cleavers, promoting the strand scission of plasmid DNA. Finally, studies were performed to quench Trp and Tyr fluorescence residues in the human serum albumin protein. The combination of fluorescence techniques indicated a static mechanism for **23** and **24**, and was complemented by molecular docking results.

These copper‐based compounds were evaluated for their cytotoxicity against HeLa, A549, and MDA‐MB‐231 cancer cell lines. All complexes exhibited significant cytotoxicity against HeLa cells, with IC_50_ values of 2.04 ± 0.04 and 1.86 ± 0.05 μM for complexes **23** and **24**, respectively (Figure 4). In order to verify if apoptosis is the main pathway involved in the mechanism of these compounds, further mechanistic investigations were performed using a synthetic tetrapeptide, Asp‐Glu‐Val‐Asp (DEVD), labeled with p‐nitroaniline (p‐NA). Complexes **23** and **24** showed approximately 1.5‐fold increase in caspase‐3 activation, indicating their ability to induce apoptosis via caspase activation. To gain more insight into their mechanism, fluorescence images revealed significant damage to actin filaments after treatment with these compounds, suggesting their capacity to affect cytoskeletal structure in HeLa cells.

Manganese(III)–salen compounds were also investigated by Khatun et al. [96]. The authors synthesized a Mn(III) complex (**25**) (Figure 4) and confirmed its structure using XRD. The complex exhibited significant anticancer activity against MCF‐7 cells, with an IC_50_ value of 6.93 μM. DNA interaction studies, including the Hoechst competitive fluorescence assay, viscosity measurements, and circular dichroism (CD) spectroscopy, indicated a minor groove binding mode. Furthermore, complex **25** effectively suppressed cancer cell growth in both 2D monolayers and 3D mammosphere models as indicated by data obtained from colony formation assays, wound‐healing assays, and cell growth inhibition analyses.

Keshavarzian et al. [97] synthesized and characterized Co(III) and Cu(II) complexes (**26** and **27**) (Figure 4). They evaluated the cytotoxic activities of these complexes against MCF‐7 and A549 cancer cell lines. The Cu(II) complex exhibited higher cytotoxicity against A549 tumor cells compared to MCF‐7 cells. Furthermore, this compound was shown to induce apoptosis through the intrinsic mitochondrial pathway, underscoring its potential as a promising anticancer agent. Moreover, DNA binding studies confirmed that both complexes interact with DNA via an intercalative mode.

The influence of different heterocyclic ligands (py, bipy, and phen) in the chemical structures of **28**–**30** (Figure 4) was investigated by Ghasemi et al. [98]. Preliminary in vitro studies of these complexes were assessed by 2D monolayers of human colorectal HCT116, A549, and MCF‐7 cancer cell lines. Notably, Cu(II) complex **29** demonstrated the highest cytotoxicity, exhibiting IC_50_ values of 4.16 μM (A549), in comparison to 7.82 μM (HCT116) and 7.82 μM (MCF‐7), surpassing the efficacy of 5‐FU and oxaliplatin drugs. Although the presence of bipy plays an important role in the cytotoxic profile of **29**, additional results are necessary to explore the mechanism of action of these compounds.

Talebi et al. [99] synthesized and characterized Ni(II), V(IV), and Cu(II) complexes **31**–**33** (Figure 4) using IR, UV–vis, cyclic voltammetry, and XRD. The complexes demonstrated cytotoxicity against HT‐29 cancer cells, with the V(IV) complex **33** showing superior activity (IC_50_ = 10.83 μM) compared to 5‐FU (IC_50_ = 61.23 μM). Molecular docking studies revealed that these complexes interact with the COX‐2 enzyme (PDB: 1CX2) through van der Waals forces, hydrophobic interactions, and hydrogen bonding, suggesting that the V(IV) complex **33** exhibits potent anticancer activity against HT‐29 cells, likely through COX‐2 inhibition mediated by hydrophobic and hydrogen‐bond interactions with the 1CX2 receptor.

The anticancer activity of Cu(II), Zn(II), and Mn(II) complexes **34–36** (Figure 4) was evaluated by Gai et al. [100] against A549, MCF‐7, and HeLa cells. In vitro results revealed **34** as the most promising cytotoxic complex, with IC_50_ equal to 3.93 μM against A549 cancer cells. This value is almost five‐ and ninefold lower than those obtained for the analogous **35** and **36**, respectively, in the same cell line. After confirming its higher selectivity (SI = 2.60), the complex was chosen for further biological investigations. The potential to inhibit migration and invasion in A549 cells was evaluated by wound‐healing and transwell assays. The results revealed a decrease in both migrated area and number of invaded cells after treatment with **35** and **36** during 24 h, suggesting that these complexes could be effective antimetastatic agents. Furthermore, flow cytometry experiments were conducted to gain new insights into the mechanism of action for **35**. Whereas JC‐1 dye was used to investigate the status of the mitochondria by evaluating the MMP, Annexin V/PI double‐staining assay was employed to monitor the cell dead population. The results revealed that **35** induced apoptosis driven by mitochondrial dysfunction. Additional experiments indicated this complex as an ROS‐inducing agent that promotes DNA damage in lung cancer cells. The in vivo efficacy of this compound was confirmed in mice bearing A549 tumors, revealing an inhibition rate of 46% after treatment with the metal complex. Histological analysis showed no pathological alterations, indicating the biocompatibility and biosafety of Zn(II) complex **35**.

**FIGURE 5:**
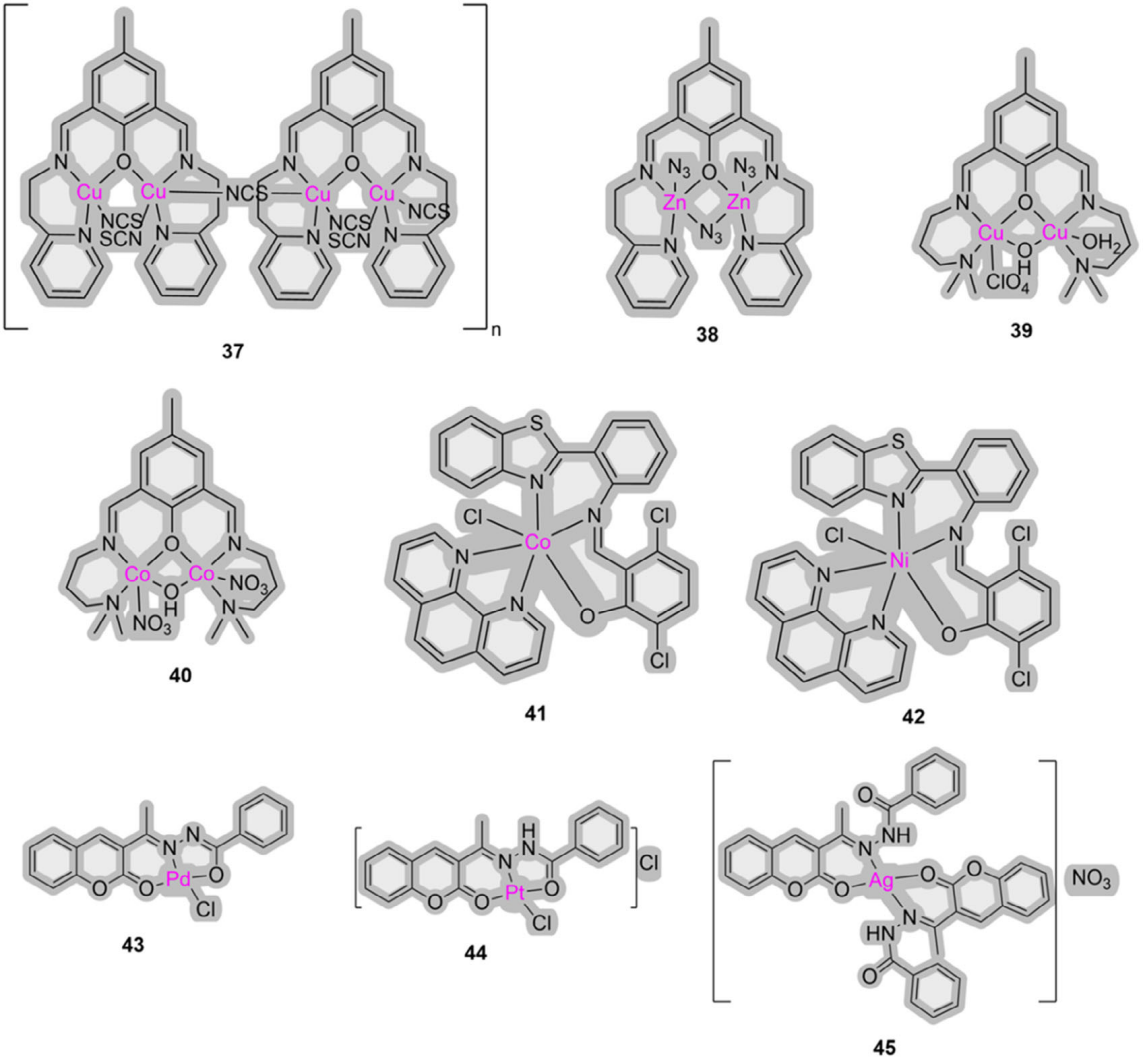
Schiff base complexes investigated as anticancer agents.

In order to improve the cytotoxicity of these metal‐based compounds, Jahromi et al. [101] studied different binuclear complexes (Figure 5). The authors synthesized and characterized Cu(II) and Zn(II) complexes **37**–**38** using IR, UV–vis, and XRD. These species were evaluated for their cytotoxic effects against AGS, MCF‐7, and PC3 tumor cells as well as NIH/3T3 normal cells. The Cu(II) complex **37** showed higher cytotoxicity and selectivity against AGS cells (IC_50_ = 29.49 ± 2.18 μM, selectivity index SI = 1.43) than the Zn(II) complex **38** while showing minimal toxicity toward NIH/3T3 cells. DNA binding studies were determined by viscosity and CD measurements, indicating that both complexes interact with DNA through intercalation. Additionally, flow cytometry analysis confirmed that the complexes induce apoptosis, suggesting their potential as selective anticancer agents.

Similar complexes were investigated by Amirian et al. [102]. In the same line, Cu(II) and Co(II) complexes **39** and **40** (Figure 5) were characterized by using FT‐IR, UV–vis, NMR, molar conductivity, and XRD. The anticancer activity of these complexes was evaluated against MDA‐MB‐231, A549, and HeLa cancer cell lines. The Co(II) complex **40** exhibited superior activity against A549 cells (IC_50_ = 16.23 ± 2.97 μM) compared to cisplatin (IC_50_ = 24.51 ± 2.87 μM) and demonstrated favorable selectivity (SI = 2.98 vs. cisplatin's SI = 3.43) while showing low toxicity toward normal NIH/3T3 fibroblasts. DNA interaction studies confirmed an intercalative binding mode for both complexes. Mechanistic studies revealed that the Co(II) complex **40** induced apoptosis in A549 cells through ROS generation and S‐phase cell cycle arrest, highlighting its potential as a targeted anticancer agent.

Ibrahim et al. [103] reported Co(II) and Ni(II) complexes (**41** and **42**) (Figure 5), which were characterized using UV–vis, FT‐IR, NMR, and mass spectrometry. Analogous compounds containing two Schiff bases as ligand were also obtained. The cytotoxicity of these complexes was evaluated against HepG2, HeLa, and A549 cancer cells. The studies revealed that **41** and **42** complexes exhibited higher cytotoxicity toward the cell lines investigated, but were less cytotoxic than the reference drug 5‐FU. The complexes demonstrated DNA intercalation activity and induced cancer cell death through an apoptosis‐mediated pathway.

Different Pd(II), Pt(II), and Ag(I) complexes containing 3‐acetylcoumarin benzoylhydrazone Schiff base were synthesized and characterized by Elsayed et al. [104] (**43**–**45**) (Figure 5). The stability of these compounds was studied in different conditions. Despite the high stability of **44** during 48 h, UV–vis experiments in DMSO/buffer solution suggested a solvent exchange for **43** and **45**. The cytotoxicity of these complexes was evaluated against HeLa and MCF‐7 cancer cell lines, as well as the noncancerous WI‐38 cell line at 24 and 48 h. Both Pd(II) **(43)** and Pt(II) **(44)** complexes exhibited superior cytotoxicity and selectivity toward MCF‐7 and HeLa cells while showing lower toxicity toward WI‐38 cells, highlighting complex **43** (SI = 5.31 and 4.62 for 24 h and 48 h, respectively). The DNA binding constants (*K*_b_) for these compounds were determined to be 4.71 × 10^6^ and 2.58 × 10^6^ M^−1^ for **43** and **44**, respectively, indicating strong interactions with the biomolecule. Also, the interaction between these complexes and bovine serum albumin is driven by non‐covalent interactions such as electrostatic and hydrogen bonding. Mechanistic studies of Pd(II) and Pt(II) complexes (43 and 44) revealed that these compounds act at different phases of the cell cycle. Furthermore, Annexin V/PI staining confirmed the increase in the apoptotic cell population upon treatment with **43** and **44**.

**FIGURE 6:**
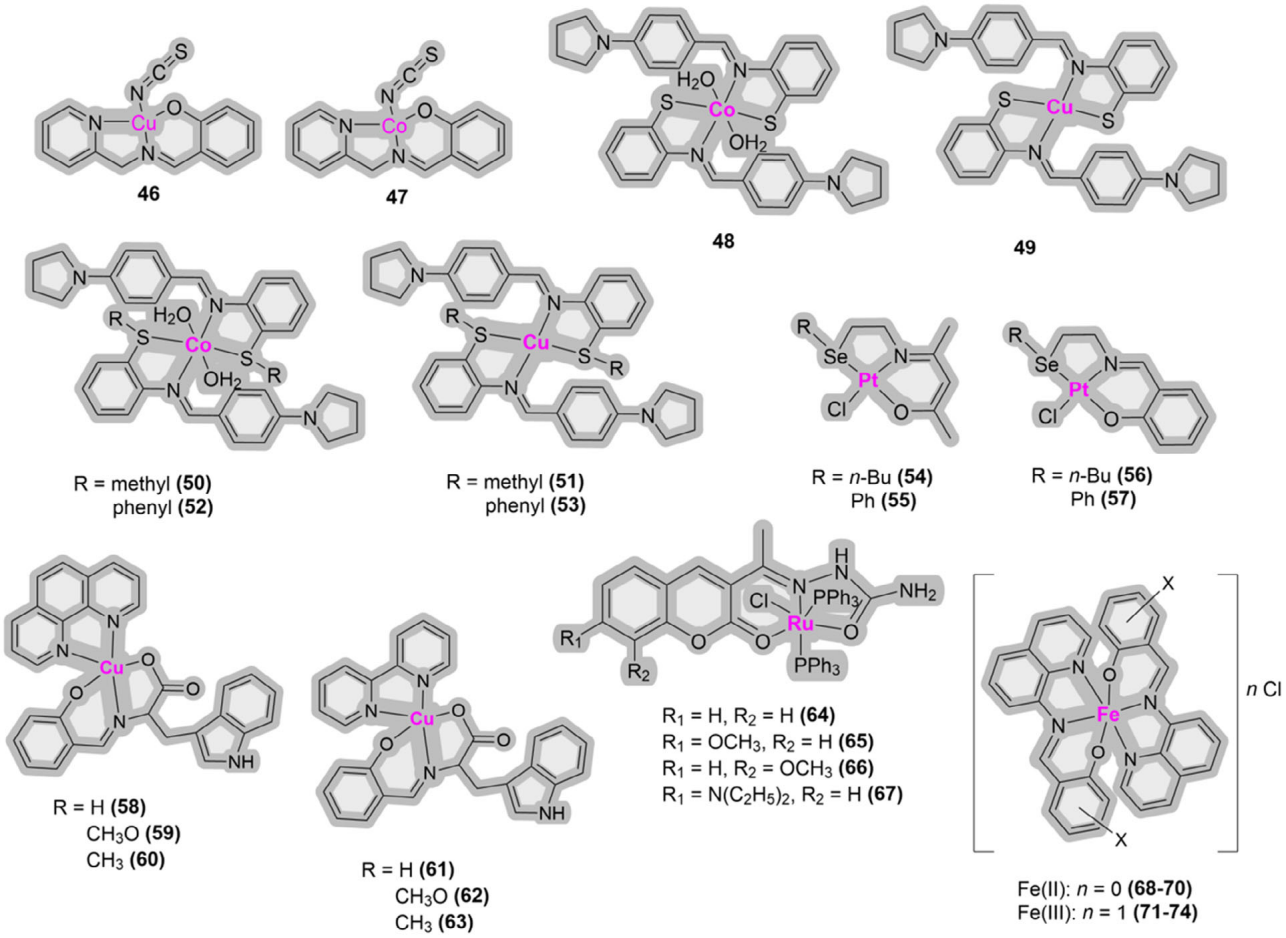
Schiff base complexes investigated as anticancer agents.

Satapathi et al. [105] reported the synthesis and characterization of Cu(II) and Co(II) complexes (**46** and **47**) (Figure 6) using several spectroscopic techniques. The cytotoxicity of these complexes was evaluated against human tumor cells (HeLa) and normal HEK 293 cells. The Cu(II) complex **47** (IC_50_ = 24.51 ± 2.73 μM) exhibited superior cytotoxicity toward HeLa cells compared to the Cu(II) complex **47**, while showing lower cytotoxicity than cisplatin (IC_50_ > 18.79 ± 2.45 μM) and minimal toxicity toward normal HEK 293 cells. DNA binding assays revealed that both complexes interact with DNA via groove binding. Additionally, AO/PI (acridine orange/propidium iodide) staining confirmed that the **46** and **47** induce apoptosis as the mechanism of cell death in HeLa cells.

Additional Co(II) and Cu(II) complexes have been studied biologically (**48**–**53**) (Figure 6). Chinnasamy et al. [106] evaluated them toward MDA‐MB‐231 and HeLa cancer cell lines. Among them, the Cu(II) (**53**) complex demonstrated the highest efficacy, with IC_50_ values of 56.99 and 39.91 µM against MDA‐MB‐231 and HeLa cells, respectively. DNA interaction studies revealed an intercalative binding mode for all complexes tested. Finally, these compounds can induce apoptosis through ROS‐mediated pathways.

Yadav et al. [107] evaluated their anticancer activity against MCF‐7 and noncancerous fibroblast cells (NIH‐3T3). Among them, the complex **57** (Figure 6) exhibited the highest potency against MCF‐7 cells, with an IC_50_ value of 6.55 ± 0.97 µM. Studies indicated that these complexes interact with DNA through a non‐intercalative binding mechanism. Researchers also reported that these compounds induced apoptosis through the ROS generation, nuclear fragmentation, and cytoskeletal disruption.

Dong et al. [108] investigated different L‐tryptophan Schiff base copper(II) complexes (**58–63**) (Figure 6). The in vitro anticancer potential of these compounds was assessed against MCF‐7, SGC‐7901, Eca‐109, HepG2, and human skin fibroblast (HSF) cells. All complexes exhibited cytotoxicity against the tested cancer cell lines, with Cu(II) (**58**) showing the most potent activity, displaying IC_50_ values of 0.80 ± 0.03 μM (Eca‐109), 0.85 ± 0.03 μM (MCF‐7), 1.15 ± 0.06 μM (HepG2), and 0.74 ± 0.04 μM (SGC‐7901), outperforming cisplatin. In particular, the Cu(II) **58**–**63** complexes showed the highest selectivity toward Eca‐109 cells, warranting further pharmacological evaluation. DNA interaction studies confirmed that these complexes bind to DNA via groove binding or electrostatic interactions. In addition, the authors have shown that Cu(II) complex **58** promoted apoptosis in Eca‐109 cells in a concentration‐dependent manner induced by ROS generation and a decrease in MMP. Moreover, the study revealed that **58** also regulated the levels of apoptotic/autophagy proteins and, therefore, played a crucial role in these mechanistic pathways.

Nandhini et al. [109] reported studies on ruthenium complexes. Complexes **64**–**67** (Figure 6) were investigated for their anticancer profile against MDA‐MB‐231. Compounds **64** and **67** exhibited superior cytotoxicity among the synthesized complexes. DNA binding studies of the complexes revealed an intercalative binding mode. Cell death mechanisms were confirmed through cell cycle arrest assays, indicating induced apoptosis in MDA‐MB‐231 cells.

Finally, Wongsuwan et al. [110] reported a series of Fe(II) **68**–**70** and Fe(III) **71**–**74** complexes (Figure 6). These complexes exhibited cytotoxicity against the A549 cancer cell lines, with Fe(III) complex **72** showing the most potent activity. DNA interaction studies revealed an intercalation binding mode, while mechanistic investigations indicated that most complexes induced apoptosis in A549 cells via DNA binding and intracellular ROS generation.

Table 2 summarizes the DNA binding behavior and cell death mechanisms reported for Schiff base complexes across different cancer cell lines. The data highlight the diversity of interactions exhibited by these complexes, including intercalative and non‐intercalative binding modes, and their ability to induce cellular responses such as DNA damage and apoptosis.

**TABLE 2:** The DNA binding modes and cell death mechanisms of Schiff base complexes in different cancer cell lines.

Codes	Cancer cells	DNA binding mode	Death mechanism	References
**1**	A549	Intercalation	DNA damage	[88]
**2–3**	MCF‐7/A549	Intercalation	Apoptosis	[32]
**4–5**	MCF‐7/A549	Non‐intercalation	Apoptosis	[89]
**6–8**	MCF‐7/A549	Intercalation	DNA damage	[90]
**9–11**	HCT‐116/CT‐26	Groove binding	Apoptosis	[91]
**12–14**	MCF‐7/HepG2	Intercalation	nd	[92]
**15–17**	HCT‐116/HepG2	nd	nd	[93]
**18–21**	DLD‐1/MDA‐MB‐231	nd	nd	[94]
**22–24**	HeLa/A549/MDA‐MB‐231	Intercalation	Apoptosis	[95]
**25**	MCF‐7	Minor groove	nd	[96]
**26–27**	MCF‐7/A549	Intercalation	Apoptosis	[97]
**28–30**	A549/HCT‐116/MCF‐7	nd	nd	[98]
**31–33**	HT‐29	van der Waals and hydrophobic interactions	nd	[99]
**34–36**	A549/MCF‐7/HeLa	Intercalation	nd	[100]
**37–38**	MCF‐7/AGS/PC3/NIH/3T3	Intercalation	Apoptosis	[101]
**39–40**	MDA‐MB‐231/A549/HeLa/NIH/3T3	Intercalation	Apoptosis	[102]
**41–42**	HepG2/HeLa/A549	Intercalation	nd	[103]
**43–45**	WI38/HeLa/MCF‐7	Intercalation	Apoptosis	[104]
**46–47**	HeLa/HEK293	Minor groove	Apoptosis	[105]
**48–53**	MDA‐MB‐231/HeLa	Intercalation	Apoptosis	[106]
**54–57**	MCF‐7/NIH‐3T3	Minor groove	Apoptosis	[107]
**58–63**	MCF‐7/SGC‐7901/Eca‐109/HepG2/HSF	Minor groove	Apoptosis	[108]
**64–67**	MD‐MB‐231	Intercalation	Apoptosis	[109]
**68–74**	A549	Intercalation	Apoptosis	[110]

*Note:* nd = not determined.

## 3 Conclusions and Future Perspective

Although Schiff bases have long been recognized for their extensive biological applications, particularly as potential therapeutic agents against various cancer cell lines, as discussed in this review, cancer remains a challenging and life‐threatening disease worldwide. Therefore, further research is necessary to develop new Schiff bases and their complexes with improved anticancer efficacy and fewer side effects. Schiff bases containing metals, such as palladium, platinum, copper, iron, nickel, zinc, cobalt, mercury, cadmium, manganese, ruthenium, vanadium, chromium, silver, and zirconium, are frequently used and show significant cytotoxicity against tumor cell lines. These findings are likely to motivate scientists to develop new potential therapeutic drugs. With ongoing research and development, SBMCs hold the potential to transform cancer treatment by offering more targeted, effective, and less toxic therapeutic options. The future of cancer therapy involving SBMCs is auspicious, offering significant opportunities to improve patient outcomes while reducing treatment‐related side effects.

## Author Contributions

**Atif Ahmad**: writing – original draft, review and editing. **Anam**: writing – review and editing. **Marcos V. Palmeira‐Mello**: writing – review and editing. **Sabir Khan**: writing – review and editing. **José Clayston Melo Pereira**: writing – review and editing.

## Conflicts of Interest

The authors declare no conflicts of interest.

## Data Availability

All data are available in the text.
